# Necklace-like NiO-CuO Heterogeneous Composite Hollow Nanostructure: Preparation, Formation Mechanism and Structure Control

**DOI:** 10.1038/s41598-017-00157-0

**Published:** 2017-03-10

**Authors:** Shao Hui Xu, Guang Tao Fei, Hao Miao Ouyang, Guo Liang Shang, Xu Dong Gao, Li De Zhang

**Affiliations:** Key Laboratory of Materials Physics and Anhui Key Laboratory of Nanomaterials and Nanotechnology, Institute of Solid State Physics, Hefei Institutes of Physical Science, Chinese Academy of Sciences, P. O. Box 1129, Hefei, 230031 P. R. China

## Abstract

Composite hollow nanostructure composed by transition metal oxides are promising materials in electrochemistry, catalyst chemistry and material science. In this contribution, necklace-like NiO-CuO heterogeneous composite hollow nanostructures were synthesized by annealing Ni/Cu superlattice nanowires in air. Two kinds of morphologies including CuO nanotube linked core-shell structures and CuO nanotube linked hollow structures were obtained. The structure can be tuned easily by adjusting the relative length of Cu segments in Ni/Cu superlattice nanowires and the annealing temperature. The relative diffusion amount of Cu to Ni segments was proved to be the key factor to influence the annealed sample morphology. The formation mechanism was discussed in detail based on Kirkendal effect and high temperature oxidation of alloy. We demonstrated that hollow structure or core-shell structure is related to whether the oxidation exists only in external sites or co-exists in external and internal sites during annealing.

## Introduction

Nanostructured transition metal oxides (MO, where M is Fe, Co, Ni and Cu) are considered as promising candidates for numerous applications, such as, lithium ion batteries^1, 2^, supercapacitor^3–5^, gas sensor^6, 7^, electrocatalyst^8, 9^ and so on. In order to meet different requirements of applications, MO nanostructures with various morphologies including nanowires^10^, nanotubes^11^, nanorods^12^, nanoflakes^13^ and hollow nanospheres^14^, have been developed. Among all of them, hollow nanostructure has attracted intensive research interests owing to its special structural features including high surface area, high surface reaction activity and fast diffusion ability, which are beneficial to the improvement of the catalytic performance, gas sensing, as well as the battery properties^15^. For example, the hollow NiO hemisphere gas sensor exhibited two times higher gas response than that of pristine NiO thin film in C_2_H_5_OH gas detection^7^, which attributed to its low density nature and high surface permeability. In addition, the polycrystalline CuO hollow structures as anode materials for lithium-ion batteries showed a high initial discharge capacity of 1503.9 mA h g^−1^ with the average Coulombic efficiency of ~97.0%^16^. NiO hollow spheres presented much better cycling performance than the solid spheres, maintaining as high as 560 and 490 mA h g^−1^ of discharge/charge capacities after 45th cycles, respectively^15^. These reported improvements in electrochemical performances indicate that the hollow nanostructure has huge superiority in many applications.

So far, researches on MO hollow nanostructure are mainly focused on the nanotubes and hollow nanoparticles of single component. Through oxidation of metal at a certain temperature, kinds of nanotubes and hollow nanoparticles, such as CuO, NiO, ZnO and so on^17–23^ have been prepared based on Kirkendall effect^24–28^, and the reaction mechanisms have been also discussed in detail^29–32^. However, to the best of our knowledge, very few reports have been reported on the fabrication of heterogeneous composite hollow nanostructure. It is generally thought that the heterogeneous composite architecture can make use of the advantages of both components and thus display fascinating synergetic properties or multi-functionalities. For instance, because of the low cost and environmental compatibility of CuO, as well as the high theoretical specific capacitance of NiO, hierarchical NiO/CuO nanocomposite was considered as a promising candidate for supercapacitor^33, 34^. Moreover, novel properties may be created as an electron potential landscape would be formed at the interface, i.e., so called bandstructure engineering, when different MO components are combined. Therefore, realizing the synthesis and structure control of heterogeneous composite hollow nanostructure is quite demanded.

Herein, one dimensional NiO-CuO heterogeneous composite hollow nanostructures were prepared by annealing Ni/Cu superlattice nanowires in air. By changing the segment length of Ni/Cu superlattice nanowires and the annealing temperature, we studied the morphology evolution of the heterogeneous composite hollow nanostructure as well as the corresponding influencing factors. The formation mechanism of heterogeneous composite hollow nanostructure was discussed in detail. Our work provides a facile approach to fabrication of the heterogeneous composite hollow nanostructures, and shed light on the design and controllable synthesis of the functional heterogeneous composite hollow nanostructures.

## Results

### Morphology Characterization

**Figure 1:**
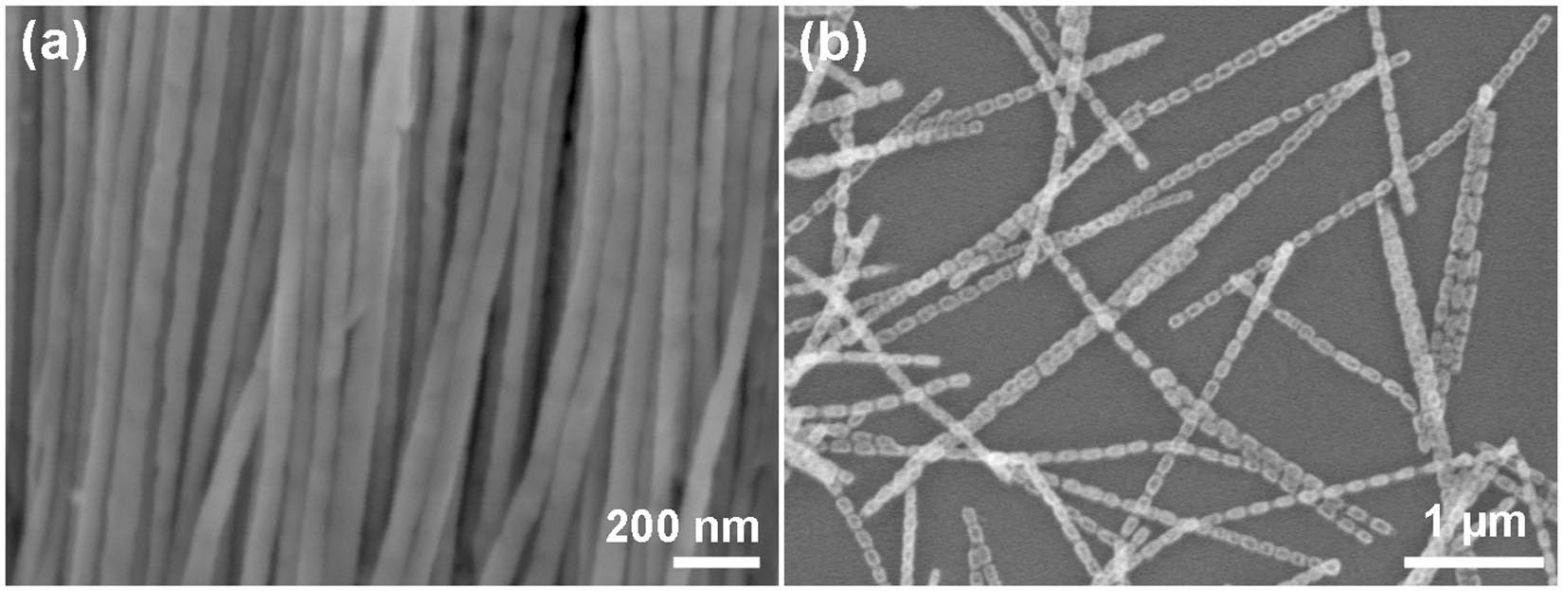
Morphology before and after annealing. FE-SEM images of (**a**) Ni/Cu nanowires arrays released from the AAO template and (**b**) after annealing at 700 °C for 2 h in air.

Figure 1a shows the FE-SEM image of Ni/Cu nanowires arrays released from AAO template completely, in which the nanowires have smooth surface and uniform diameter. After annealing at 700 °C for 2 h in air, the smooth and uniform nanowires are transformed to a fascinating morphology, with necklace-like structure consisting of periodic arranged nanoparticles (Fig. 1b). In order to better understand the morphology evolution of Ni and Cu segments, Ni/Cu samples with different segment lengths were further investigated.

**Figure 2:**
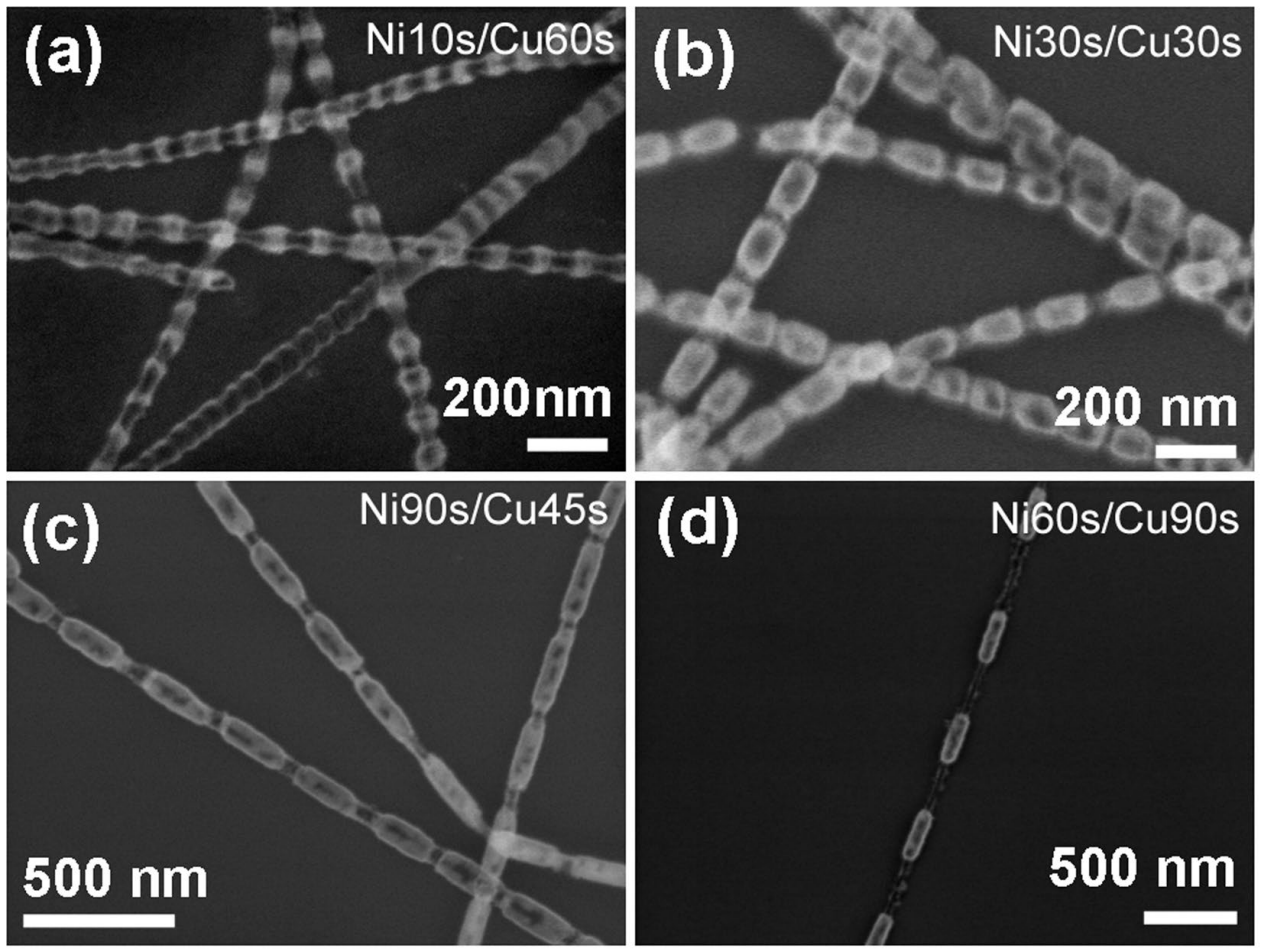
Morphologies of annealed samples with different segment lengths. FE-SEM images of the four kinds Ni/Cu samples after annealing at 700 °C for 2 h in air: (**a**) Ni10s/Cu60s; (**b**) Ni30s/Cu30s; (**c**) Ni90s/Cu45s and (**d**) Ni60s/Cu90s.

Figure 2 shows the FE-SEM images of Ni/Cu nanowires with different segment lengths after annealing at 700 °C for 2 h in air. Figure 2(a–d) correspond to the samples of Ni10s/Cu60s (meaning that the deposition times for Ni and Cu segment within one period are 10s and 60s, respectively, similarly herein after), Ni30s/Cu30s, Ni90s/Cu45s and Ni60s/Cu90s, respectively. It can be seen that the morphology of nanowires after annealing is roughly the same as that shown in Fig. 1b, except for the different periodic lengths. After carefully analysing the segment length of these nanostructures, the thicker parts in each nanowire are confirmed to be the original Ni segments and the approximate transparent tubular parts are the original Cu segments.

### Structure and Composition Characterization

**Figure 3:**
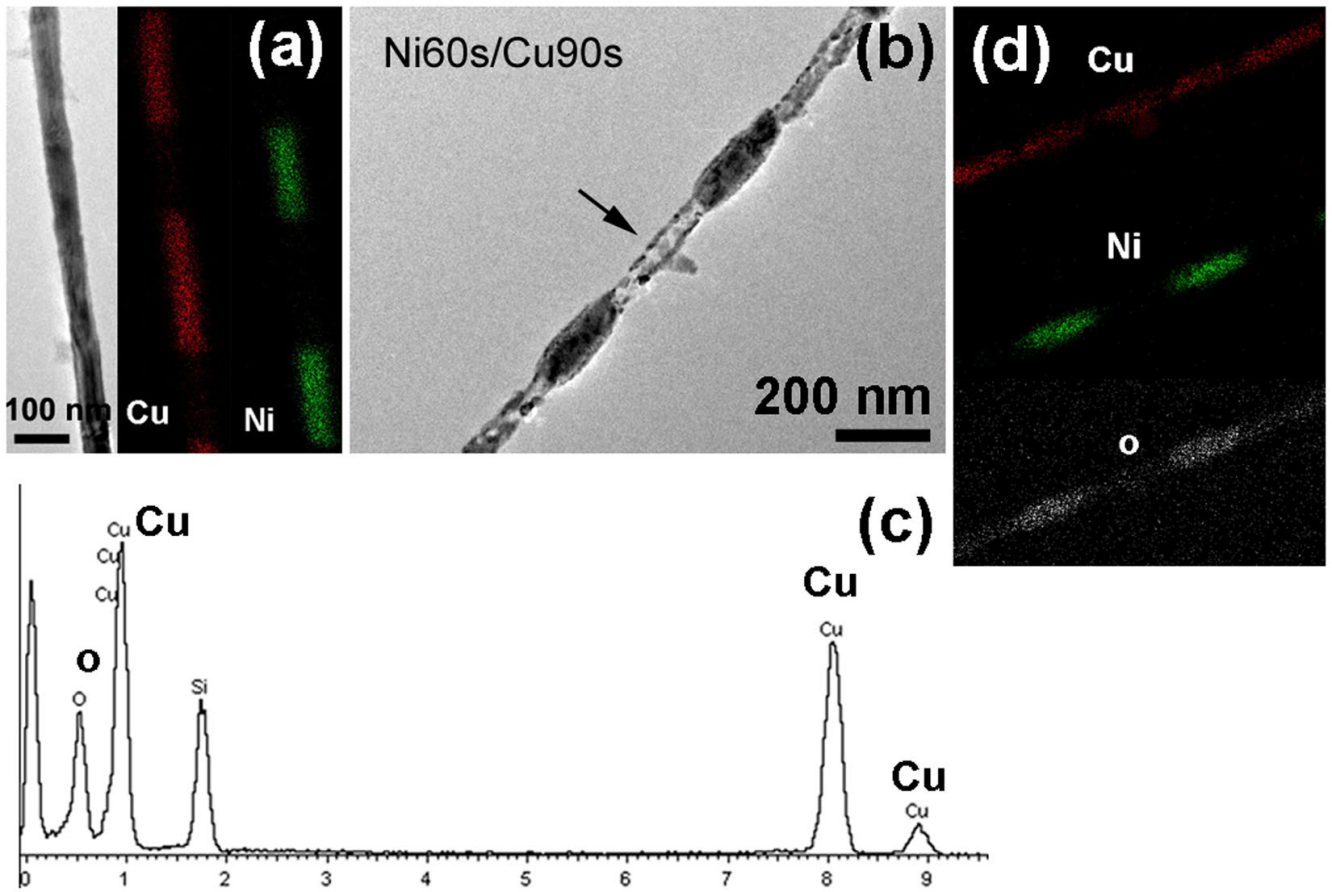
Characterization results of Ni60s/Cu90s sample. (**a**) The TEM and element EDS mapping image of the single nanowire; (**b**) the same nanowire after annealing at 700 °C for 2 h in air. (**c**) EDS result of the tubular structure that has been labelled by arrow in (**b**); (**d**) EDS mapping image of the nanostructure in (**b**).

In order to study the structural and compositional changes of the annealed Ni/Cu nanowires, detailed analysis for nanowire before and after annealing was carried out with TEM. Figure 3a is the TEM image and the corresponding EDS mapping image of single nanowire of Ni60s/Cu90s sample. It can be seen that Ni and Cu elements arrange alternately along the nanowire axial direction. Moreover, the composition of each segment is almost pure and does not contain the other element. After annealing at 700 °C for 2 h in air, the morphology and composition evolution of the nanowire are shown in Fig. 3b–d. Figure 3b shows the morphology after annealing and Fig. 3c is the EDS result of the tubular structure labelled by arrow in Fig. 3b. The EDS results indicate that Cu segment has completely transformed to CuO nanotube during annealing process. Figure 3d is the EDS mapping image of the sample in Fig. 3b, which shows that the original Cu segments are now composed of two elements of Cu and O, consistent with the EDS result in Fig. 3c, and the original Ni segments are transformed to NiO, in which a small amount of Cu element is observed, indicating Cu atoms have diffused into NiO segments in the annealing process.

**Figure 4:**
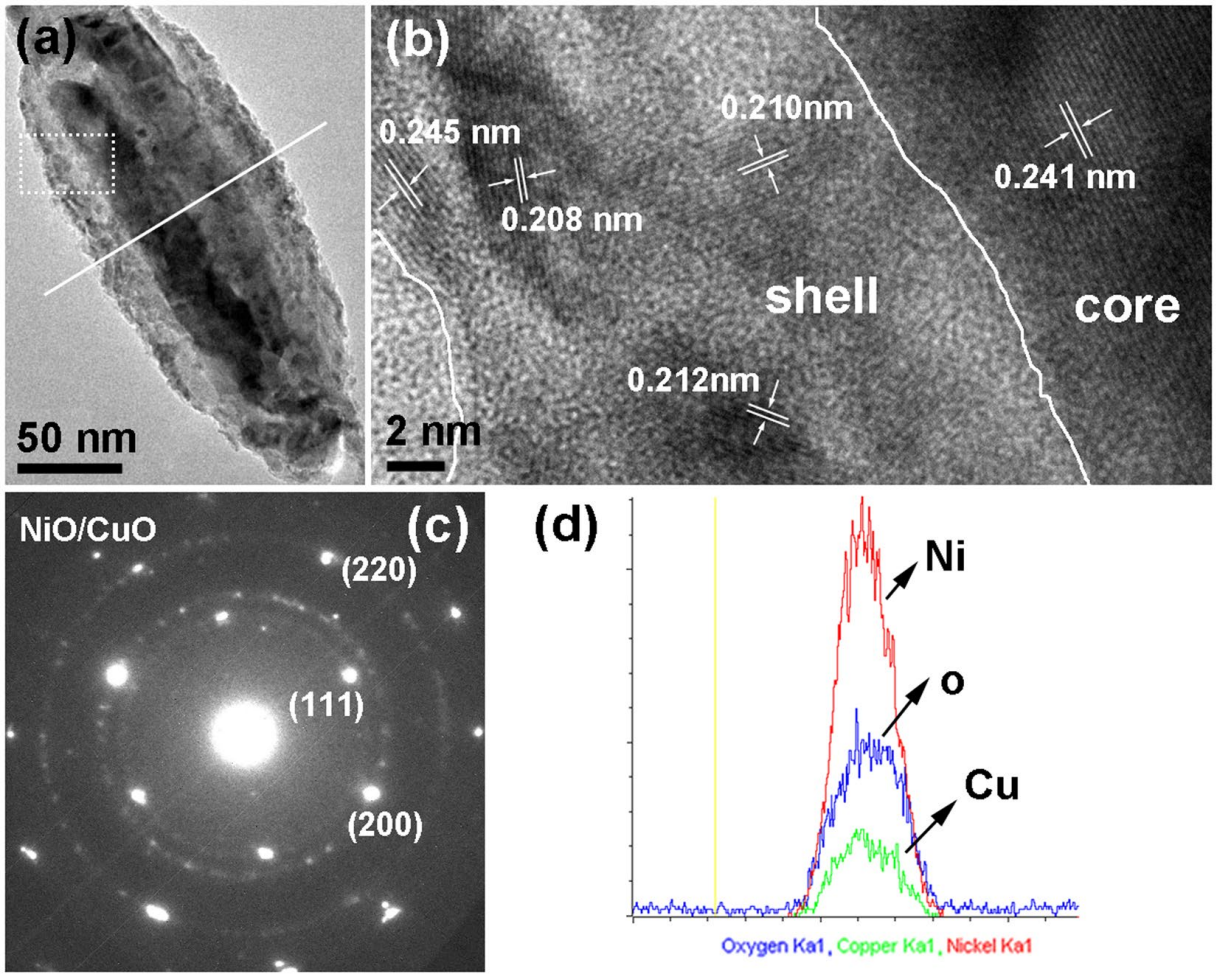
Characterization results of the core-shell structure. (**a**) The high magnification TEM image of the thick segment; (**b**) the HR-TEM image of the area labeled in (**a**); (**c**) the corresponding SAED patterns of the thick segment; (**d**) the EDS line scanning image of the thick segment along the radial orientation as the white line shown in (**a**).

Figure 4 shows the characterization results of NiO segment, from which the composition and structure can be well recognized. Figure 4a shows the high magnification TEM image of the NiO segment, which indicates Ni segment transformed into the core-shell structure. Figure 4b is the HR-TEM image of the area labelled with dotted rectangle frame in Fig. 4a. Lattice fringes indicate the shell part is of polycrystalline structure composed of small metal oxide grain with different crystal orientation. In contrary, the continuous lattice stripe can be observed in the core part and the interplanar spacing is estimated to the (111) plane of NiO or CuO, both of which possess similar interplanar spacing. The SAED patterns in Fig. 4c demonstrate NiO or CuO of face-centered cubic (FCC) structure, and no obvious diffraction spots of the metal phase can be observed. Similarly, due to the very similar interplanar spacings of the same crystal plane (200) and (220) for FCC NiO and for FCC CuO, that is, the interplanar spacings of planes (200) and (220) for NiO are 0.208 nm, 0.147 nm, respectively, and for CuO are 0.212 nm, 0.150 nm, respectively, it is difficult to distinguish them well from SAED. The appearance of diffraction rings further indicates the existence of polycrystalline ingredient, which is consistent to the HR-TEM observation (Fig. 4b). Figure 4d is the EDS line scanning image of the thick segment along the white line shown in Fig. 4(a), which shows that the shell part is NiO and CuO mixture, and the core part is rich in NiO. From the above we can come to the conclusion that the segments have been fully oxidized and transformed into corresponding oxide completely after annealing.

**Figure 5:**
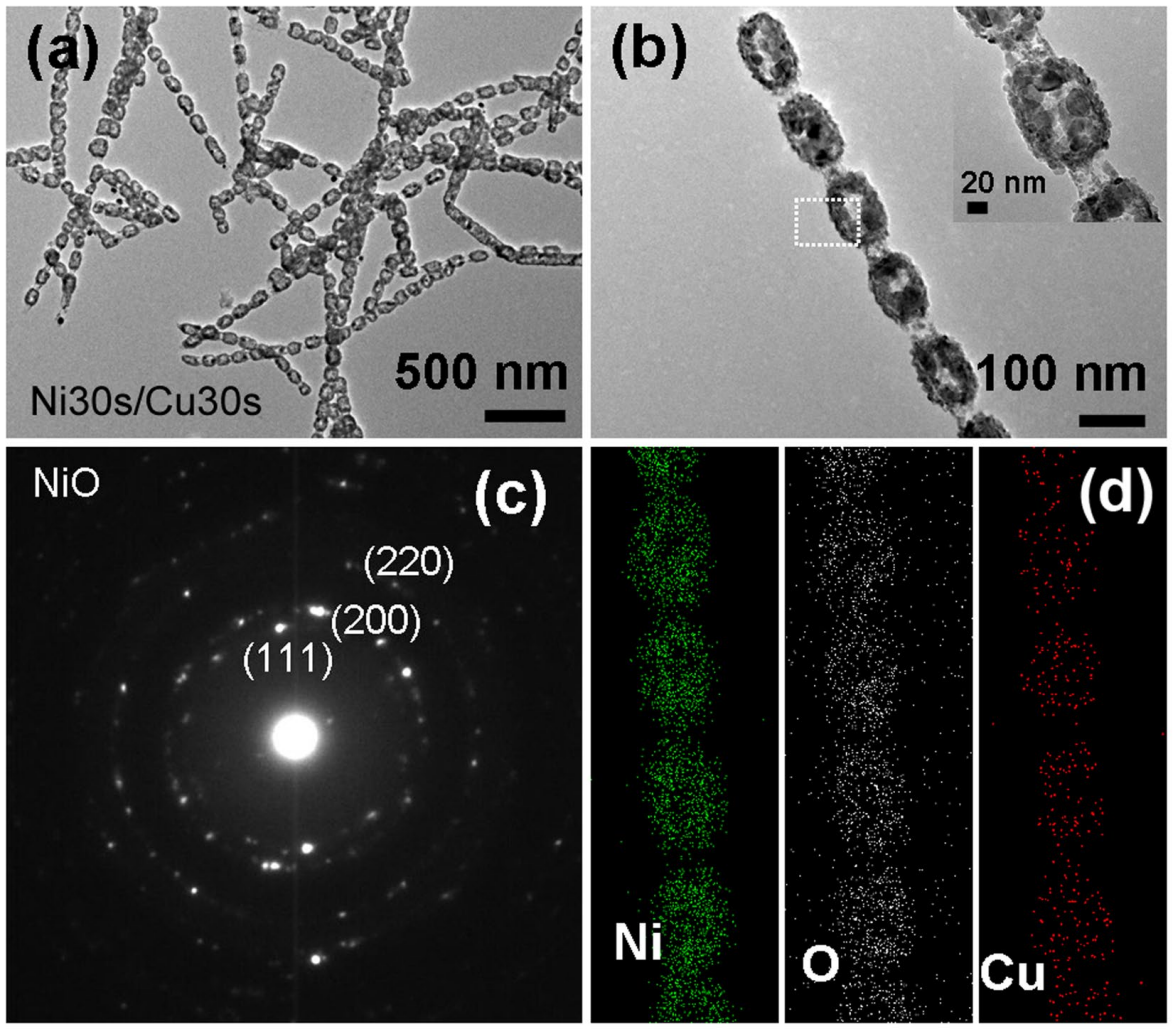
Characterization results of Ni30s/Cu30s sample. (**a**) TEM image of sample after annealing at 700 °C for 2 h in air; (**b**) a single chain after annealing and the inset is the magnified TEM image; (**c**) SAED pattern for the hollow particle area labeled in (**b**); (**d**) EDS element mapping image of the single hollow particle chain in (**b**).

The structure and composition of the annealed Ni30s/Cu30s sample with shorter Cu segments are also examined, as shown in Fig. 5. It is found that, Ni segments are converted to hollow ellipsoid (Fig. 5a), which is quite different from that of Ni60s/Cu90s sample with longer Cu segments (Fig. 3). Figure 5b shows the TEM image of single hollow ellipsoids chain, and the inset is the enlarged image. The Cu segment has transformed to tube structure, similar to previous results for Ni60s/Cu90s sample, except that some collapses are observed. For the original Ni segment, we may note the location of the nanoscale holes in the hollow particle deviate from the center part and the hollow structure does not have uniform shell thickness. The SAED pattern (Fig. 5c) for the hollow particle indicates that it has been oxidized completely. EDS mapping (Fig. 5d) for the single hollow particle chain in Fig. 5b shows the hollow particle mainly consist of Ni, O, and a little Cu element. The corresponding sum spectrum (Fig. S1) for element EDS mapping demonstrates that the atom ratio of Ni element and Cu element is 91.4% and 8.6%, respectively. All these evidences shown in Fig. 5c,d, make us believe that the original Ni segments in Ni30s/Cu30s sample have transformed to NiO hollow structure with a little CuO in them after annealing.

### Structure Regulation

**Figure 6:**
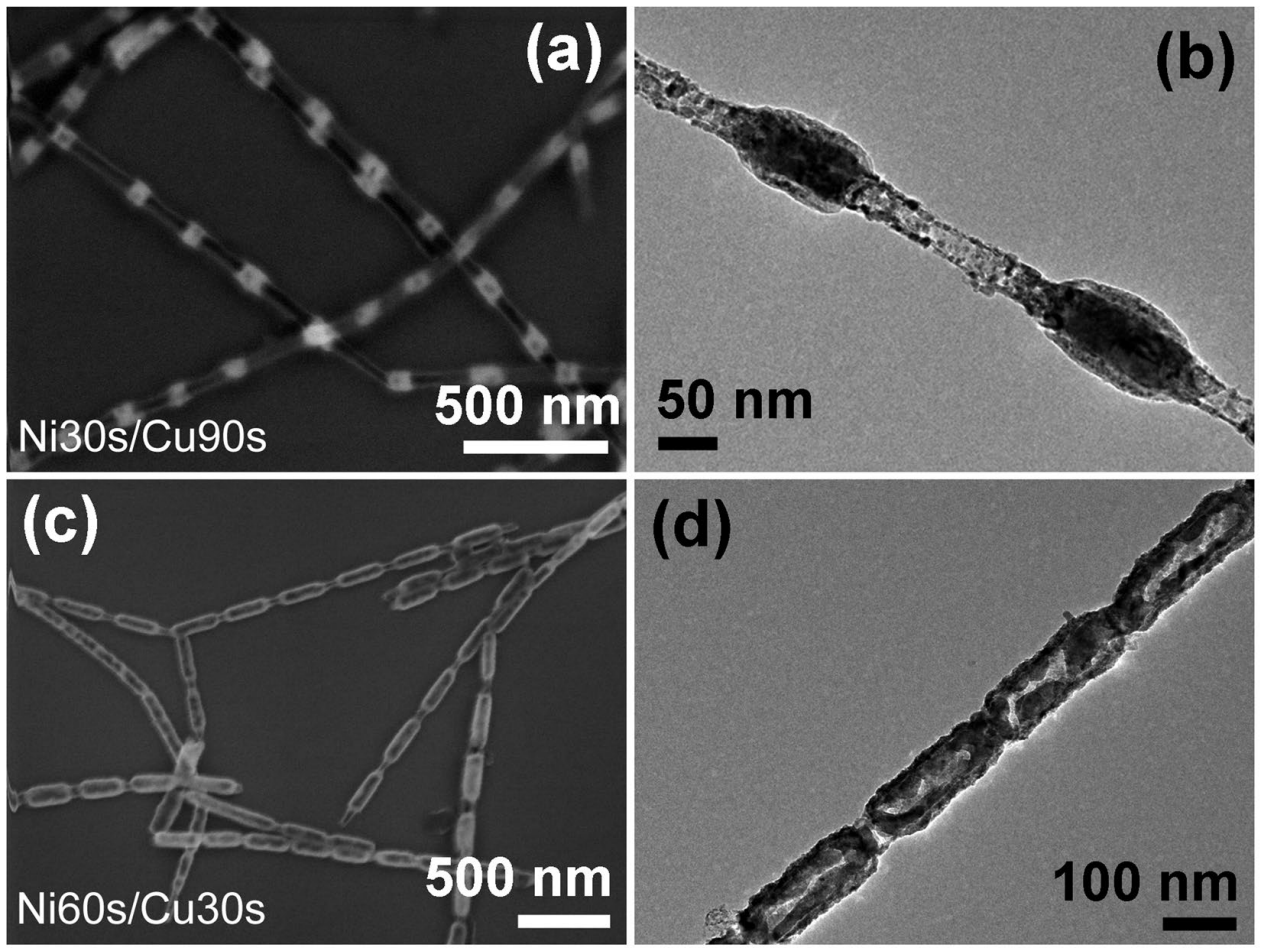
Morphology characterization with changes of Cu segment lengths. The SEM image (**a**) and TEM image (**b**) of sample Ni30s/Cu90s after annealing at 700 °C for 2 h in air, respectively; The SEM image (**c**) and TEM image (**d**) of Ni60s/Cu30s after annealing at 700 °C for 2 h in air, respectively.

Above experiment results reveal that, after annealing, the original Ni segment displays different morphologies, and the diffusion of Cu element to Ni segments occurred. Furthermore, by changing the relative length of Cu segments, the structure regulation is achieved. When prolonged the Cu segment length based on sample Ni30s/Cu30s, and reaches to a certain value, for example Ni30s/Cu90s, Ni segments no longer converted to the hollow structure but the core-shell structure after annealing (Fig. 6a,b). In contrast, when shortened the Cu segments length based on sample Ni60s/Cu90s, and less than a certain value, for example Ni60s/Cu30s, the annealed Ni segments transformed from core-shell structure to hollow structure as shown in Fig. 6c,d. Basing on the contrastive experimental results, we can conclude that, at a certain annealing temperature, the relative length of Cu segments is a critical factor that affects the morphology evolution of Ni segments.

## Discussion

Oxidations of single Cu and Ni nanostructures such as nanoparticles or nanowires have been studied extensively^18, 20, 30^. It is reported that Cu can be oxidized to hollow nanostructure with the uniform wall thickness, the component of which is Cu_2_O when annealing temperature is 150 °C and transfers to CuO at about 300 °C; while Ni is oxidized to NiO hollow nanostructure at a higher temperature of about 500 °C with non-uniform wall thickness. It has been recognized that, the mechanism of formation of hollow oxides via oxidation is attributed to the nanoscale Kirkendall effect^18, 24^, which normally refers to a nonequilibrium mutual diffusion process through the interface of coupled materials.

### The oxidation behavior of Cu segments

For Ni/Cu superlattice nanowires here, Cu segments were oxidized to CuO tubes, which is same as reported before^20^. The formation of CuO tube can be attributed to the higher diffusion rate of Cu than that of O in cuprous oxide^18, 20^. It is reported that the outward diffusion coefficient of copper ions in Cu_2_O is 3.89 × 10^−10^ cm^2^/sec at 700 °C^35^, which is several tens of times larger than the inward diffusion coefficient of oxygen ions in the same oxide at the equal temperature^36^. The unbalanced interdiffusion would lead to the formation of vacancies^24^, which gathers along Cu/Cu_2_O interface and form a plurality of interface holes in oxidation process. The evenly distributed holes ensure that the remaining Cu can symmetrically and easily spread out, and eventually a uniform wall thickness of Cu_2_O hollow nanotube formed. At higher annealing temperature, the Cu_2_O tube would finally transfer to CuO tube^20^.

### The oxidation behavior of Ni segments

For pure Ni particle oxidation, the diffusion coefficient of Ni in NiO is 1.2 × 10^−15^ cm^2^/sec at 700 °C^37^, which is at least several orders of magnitude larger than the diffusion coefficient of O in NiO^38^. Therefore, Ni spreading outward is the main process during the oxidation process resulting in the formation of the hollow structure. For Ni segments in our Ni/Cu superlattice nanowires, two different morphologies of hollow structure and core-shell structure were observed after oxidation. In addition, Cu element was observed in NiO segments, indicating a part of Cu has diffused to Ni segments during annealing. According to the Kirkendall effect^21^, when two metals contact, diffusion at the interface will occur. At the high annealing temperature herein, the diffusion effect between metals at the interface would be intensified and can not be ignored. That is, in addition to the oxidation effect of the metal itself, the diffusion effect between metals at the interface is another major process that carries out simultaneously. It is reported that the diffusion of Cu in Ni is faster than the diffusion of Ni in Cu^39–42^, so the diffusion of Cu to Ni segment should be the main process at the Ni-Cu interface, which is consistent with our observation. Therefore, besides the oxidation of Ni itself, the effect of the diffusion of Cu to Ni segments on the oxidation behaviour of Ni segments also need be considered.

The experiment results in Fig. 6 show that the morphology evolution depends on the relative length of Cu segment in Ni/Cu superlattice nanowires. We deduce that the relative diffusion quantity of Cu to Ni segment that related to relative length of Cu is the fundamental influencing factor. With this in mind, we further studied the ratio of Ni element and Cu element in the hollow structure and the core-shell structure, respectively. Figures S2 and S3 show the EDS element mapping images and the corresponding sum spectrum of the hollow structure and core-shell structure, in which the atom ratio of Ni and Cu are 96.8%: 3.2% and 81.3%: 18.7%, respectively. It prove that the content of Cu in the core-shell structures is actually higher than that in the hollow structures obviously, that is, when the relative diffusion quantity of Cu to Ni segments is low, the hollow structure formed, and when the relative diffusion quantity of Cu to Ni segments is large, the core-shell structure formed. Based on above observation and discussion, we proposed the following oxidation mechanism of Ni segments.

### Effect of relative diffusion amount of Cu to Ni on morphology evolution

**Figure 7:**
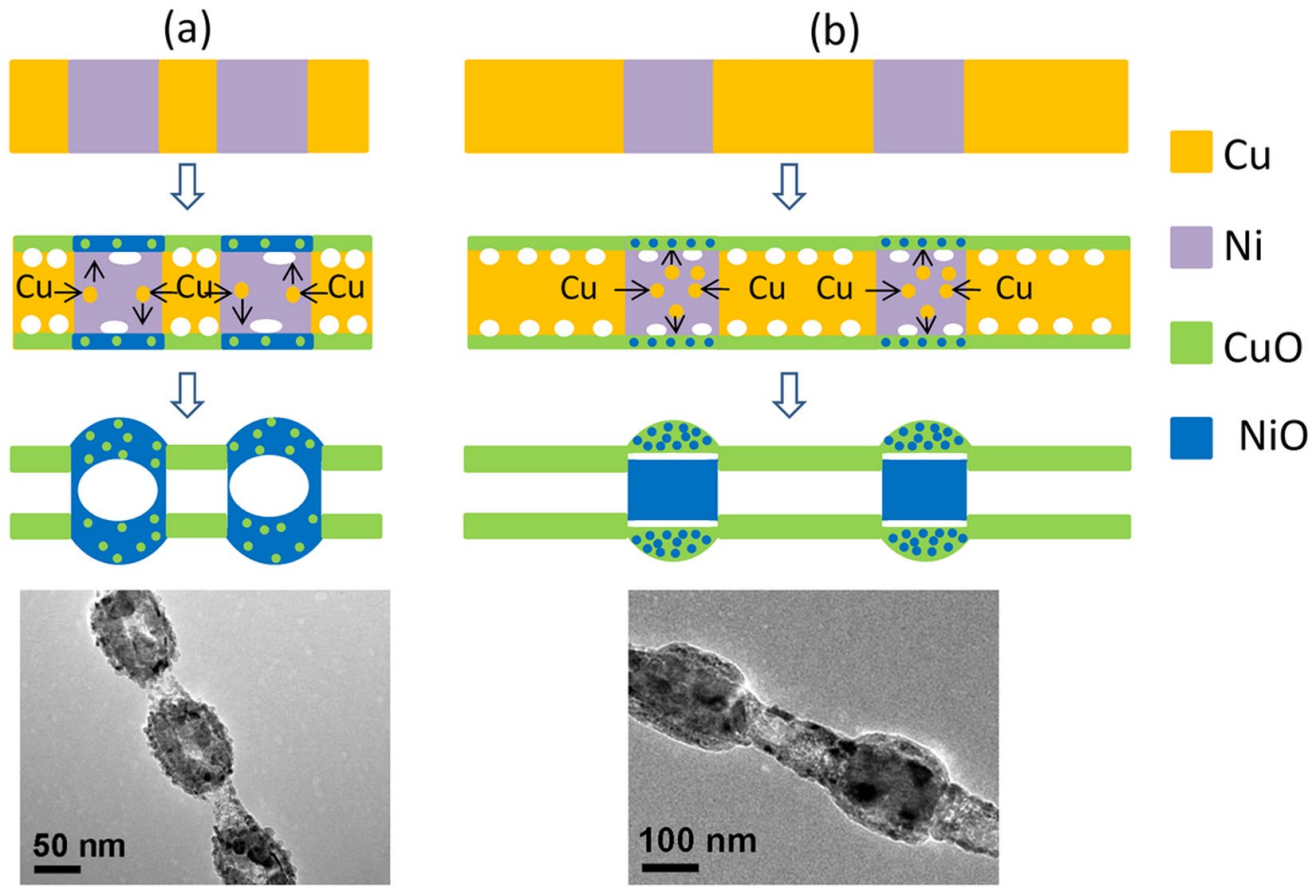
Mechanism analysis. The schematic of the morphology transformation of Ni/Cu nanowires with shorter Cu segment (**a**) and longer Cu segment (**b**) when annealing in air.

When the relative diffusion amount of Cu to Ni segment is small (Fig. 7a), the oxidation behaviour is similar to the oxidation of pure Ni, and the formation mechanism can be understand based on the nanoscale Kirkendall effect mentioned above. In this case, the external oxidation is the main process, the small amount of Cu together with Ni spread out of oxide layer and react with the outside oxygen, and the hollow structure is finally formed. The component of the hollow structure is thus NiO with a small amount of CuO dispersed in it. During the oxidation process, the vacancies have enough mobility to migrate over a long distance and aggregate inside Ni^30, 31^, which leads to the incompletely uniform shell thickness.

When the relative diffusion amount of Cu to Ni segment is large (Fig. 7b), the effect of Cu can’t be ignored. Due to that Ni and Cu are completely miscible in phase diagram of Ni-Cu alloy, the oxidation of Ni segments doped with Cu atoms can be regarded as the oxidation process of Ni-Cu alloy. The oxidation of the bulk alloy had been investigated as early as 1970 by Graham C. Wood^43^. It was reported that, high temperature oxidation of alloy is a complicated process^43–45^, the final oxidation structure is associated with the alloy composition ratio, and whether two elements are both oxidized, as well as whether two oxides are mutually compatible, etc. According to their studies, when NiCu alloy oxidized at high temperature, Ni and Cu spread out and react with outside O and produce NiO and Cu_2_O, respectively, and two oxides are virtually insoluble in each other. Cu_2_O grows faster than the NiO and protrudes further into the atmosphere and eventually overgrows the NiO. The Cu_2_O that contacts with the inner metal can partly dissociate to Cu and O to supply the inner metal Ni with atomic oxygen^43^, which leading to Ni at the alloy-oxide interface oxidized directly. NiO grows laterally to form a complete layer. In the subsequent oxidation process, the oxidation of Ni inside of the bulk alloy is mainly realized through the outward diffusion of Ni ions, as described earlier.

Herein, at the nanometer scale, Cu can almost completely diffuse out and react with oxygen due to the very short distance, and thus form an obvious shell layer in the case of the large relative diffusion amount of Cu to Ni segments. The produced Cu_2_O layer can offer sufficient O source to internal nano-sized Ni, which leads to the internal oxidation of Ni becoming another main oxidation process. Ni experiences simultaneous external and internal oxidation, which leads to the final oxidation product with an internal oxide of Ni lying beneath of a mixed shell layer of CuO and NiO, that is, core-shell structure with NiO-CuO shell and NiO-rich core here. The process of Cu_2_O dissociating to Cu and O and supplying atom oxygen to Ni can be expressed by the following chemical equation:

$${{\rm{Cu}}}_{2}{\rm{O}}+{\rm{Ni}}={\rm{NiO}}+2{\rm{Cu}}$$

The amount of change in Gibbs free energy ΔG of this reaction at 700 °C (the detailed calculation see SI) is −53.83 kJ · mol^−1^, which shows this reaction can indeed be carried out.

The mechanism analysis can be further confirmed by the experimental observations. For sample with shorter relative length of Cu segments, the oxide hollow structure is polycrystalline structure composed of a plurality of grains, which results from the outward diffusion of metal ions during oxidation process (Fig. 5c). For sample with longer relative length of Cu segments, the polycrystalline structure of oxide shell conforms to the outward diffusion process of metal ion, and the continuous lattice stripe in core part is consistent with the internal oxidation process (Fig. 4b). In addition, we might note that the nanowires have local breakage after annealing, which can be understood as follows. On the one hand, the diffusion of Cu atoms to Ni segments during the annealing process results in the emergence of a large number of vacancies at Cu side. These vacancies combine together to form pores, making the Ni-Cu interfacial contact gradually weaken. On the other hand, the oxidation may intensify the formation of voids at the interface, and eventually produce a breakage. Furthermore, as mentioned above the oxidized morphology is closely related to the relative diffusion of Cu atoms to Ni segments, which is affected by annealing process, then the oxidized morphology should also be controlled by modulating the annealing temperature. Therefore, we also studied the changes of morphology and composition with annealing temperature.

### Effect of annealing temperature on morphology evolution

**Figure 8:**
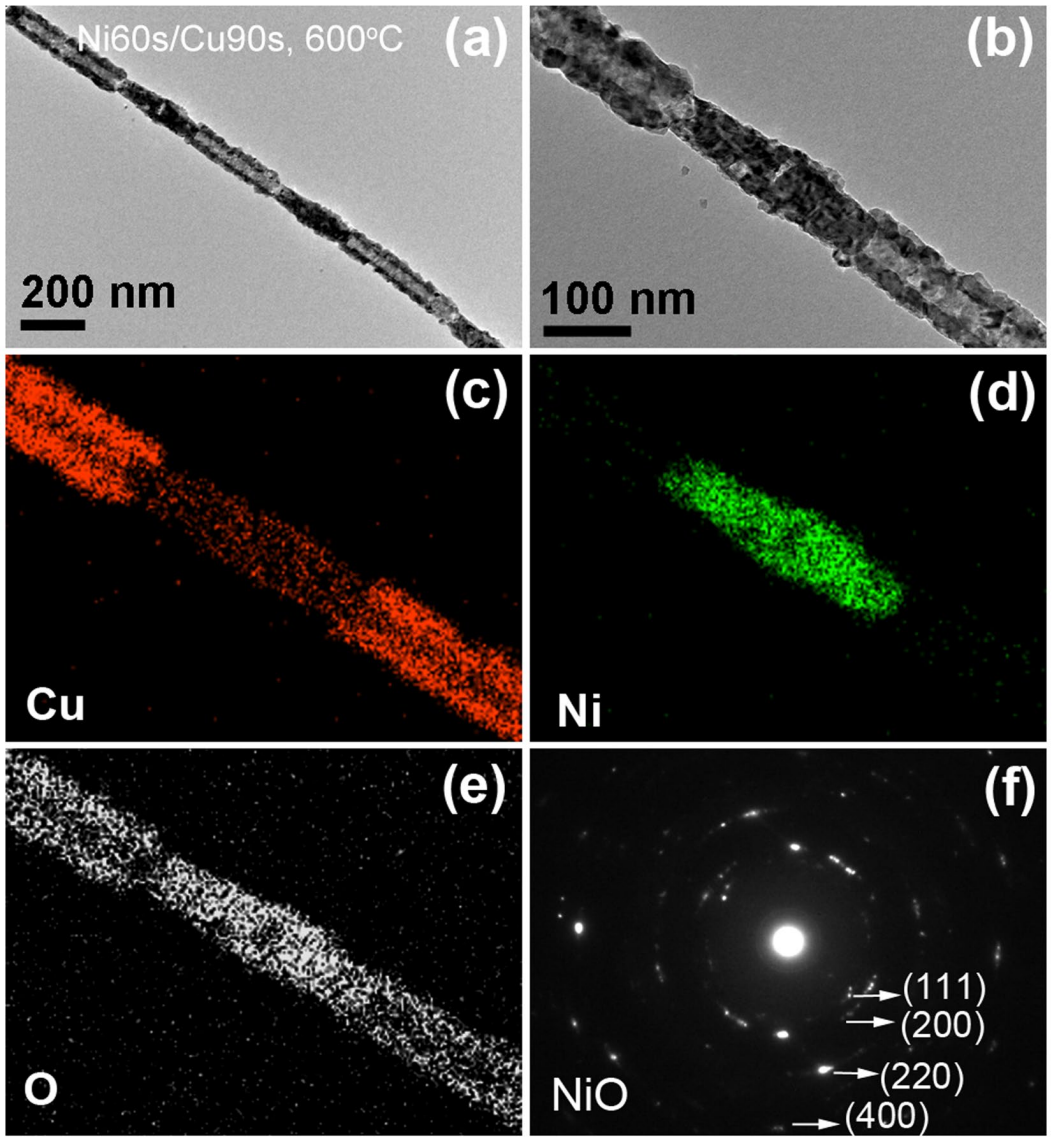
The Ni60s/Cu90s sample after annealing at 600 °C for 2 h. (**a**) TEM image of the annealed single nanowire; (**b**–**e**) magnified TEM image and the corresponding EDS elements mapping images; (**f**) SAED pattern of the NiO segment.

Figure 8 displays the characterization results of Ni60s/Cu90s sample, which has longer relative length of Cu segment, after annealing at 600 °C for 2 h. Figure 8a is the TEM image of annealed single nanowire, which shows Cu segment has changed to CuO tube with uniform wall thickness, Ni segment did not change to apparent core-shell structure like that obtained at 700 °C. From the high magnification image in Fig. 8b, we can see the oxidized Ni segment displaying the porous structure. Figure 8c–e are the corresponding EDS element mapping images of the nanostructure in Fig. 8b, a little Cu element was observed in oxidized Ni segment, indicating Cu has spread to Ni segment during the annealing process. Figure 8f is the SAED pattern of the NiO segment, in which several crystal planes corresponding to NiO has been labeled and no diffraction spots of the pure metal phase can be observed, which indicates Ni segment has been oxidized completely.

**Figure 9:**
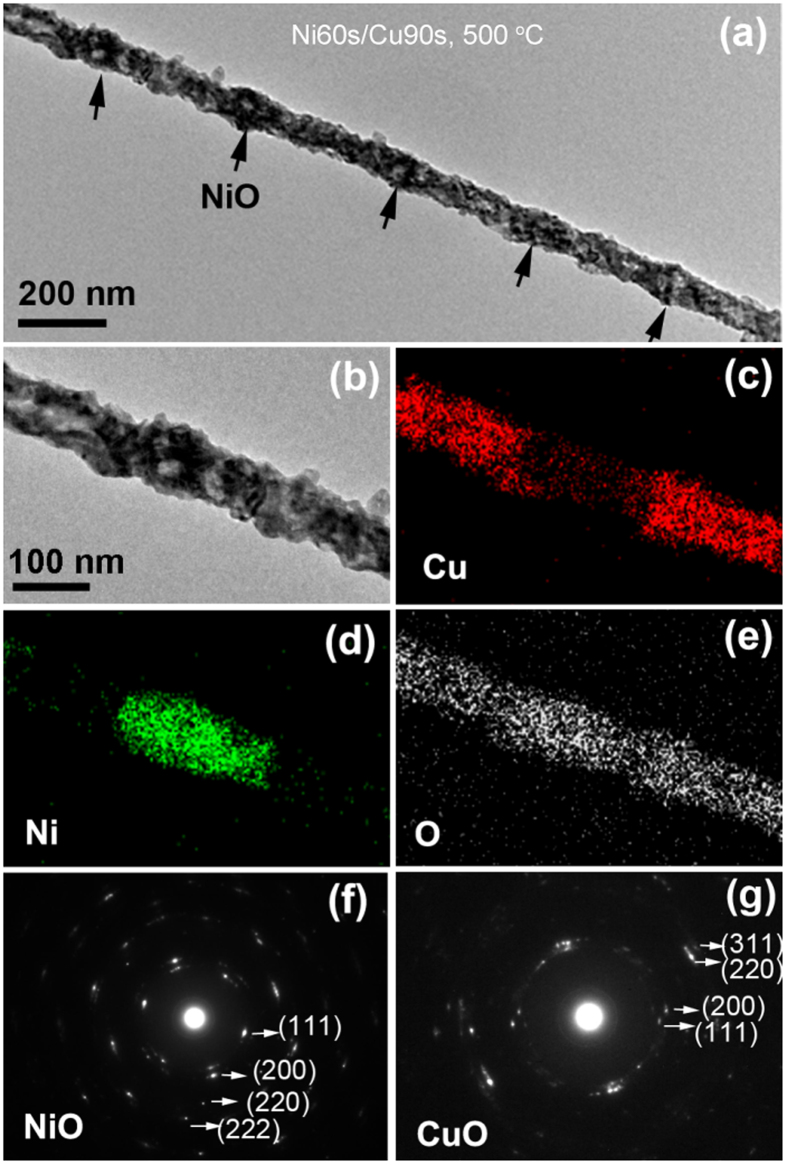
The Ni60s/Cu90s sample after annealing at 500 °C for 2 h. (**a**) TEM image of the annealed single nanowire; (**b**–**e**) the magnified TEM image and the corresponding EDS elements mapping images; (**f**) SAED pattern of the NiO segment; (**e**) SAED pattern of the CuO segment.

Figure 9 displays the characterization results of Ni60s/Cu90s nanowires after annealing at 500 °C for 2 h. In the TEM image of the annealed single nanowire (Fig. 9a), several oxidized Ni segments displayed the obvious porous structure that labeled with arrows. Figure 9b is the enlarge TEM image, the corresponding EDS element mapping images are shown in Fig. 9c–e. It can be seen that the oxidized Cu segments present a faint tubular structure with the nonuniform tube wall. A little Cu element was also observed in oxidized Ni segment, which indicating a small amount of Cu still spread to Ni segment during annealing at 500 °C. From the SAED pattern of NiO segment (Fig. 9f) and the SAED pattern of CuO segment (Fig. 9g), we can judge that Ni and Cu segments have been completely oxidized to NiO and CuO, respectively. From the above experiment results we can conclude that, for Ni/Cu nanowire with longer relative length of Cu segment, NiO segment displayed porous structure rather than the core-shell structure when decreasing the annealing temperature from 700 °C to 600 °C or 500 °C. This phenomenon can be attributed to the decreasing diffusion quantity of Cu to Ni segments with the decrease of temperature.

**Figure 10:**
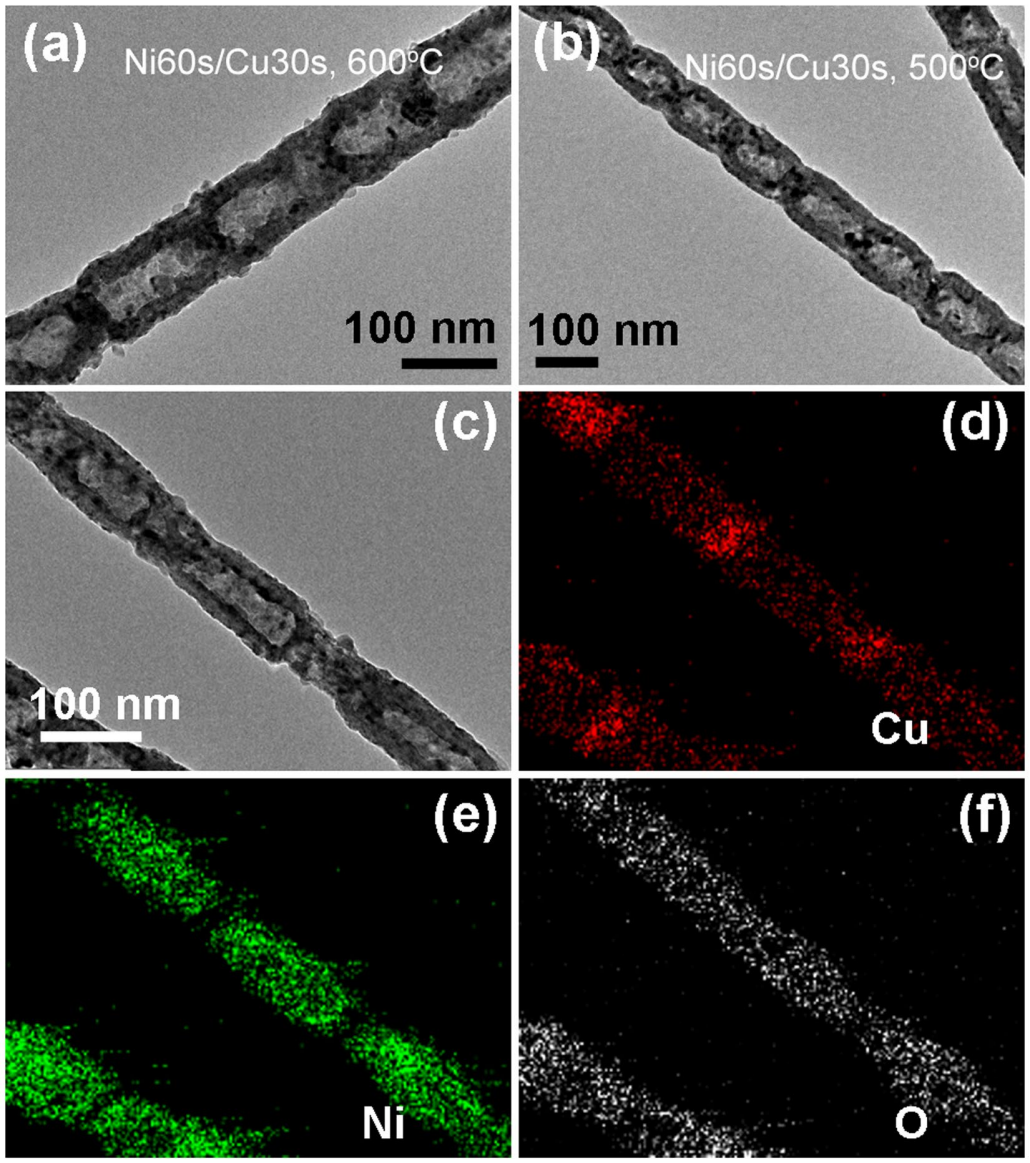
The Ni60s/Cu30s sample after annealing for 2 h. TEM images of the single annealed nanowire (**a**) at 600 °C; (**b**) at 500 °C; (**c**–**f**) the magnified TEM image and the corresponding EDS elements mapping images.

Furthermore, the morphology and composition of annealed Ni/Cu sample with shorter relative length of Cu segment at different temperatures were also studied. Figure 10a,b show the TEM images for the Ni60s/Cu30s nanowires after annealing at 600 °C and 500 °C for 2 h, respectively. It is found that all of the periodic Ni segments have been oxidized to the hollow structures no matter annealed at 600 °C or 500 °C for 2 h. Figure 10c–f give the TEM image and the corresponding EDS elements mapping images of the hollow structure obtained after annealing at 500 °C for 2 h, a little Cu element is still observed in NiO segments, which indicates Cu still spread to Ni segments. For Ni/Cu sample with shorter relative length of Cu segment, the tiny diffusion amount of Cu to Ni has almost no effect on the oxide behavior of Ni, resulting to the morphology after annealing almost unchanged.

### Component analysis at the interface

**Figure 11:**
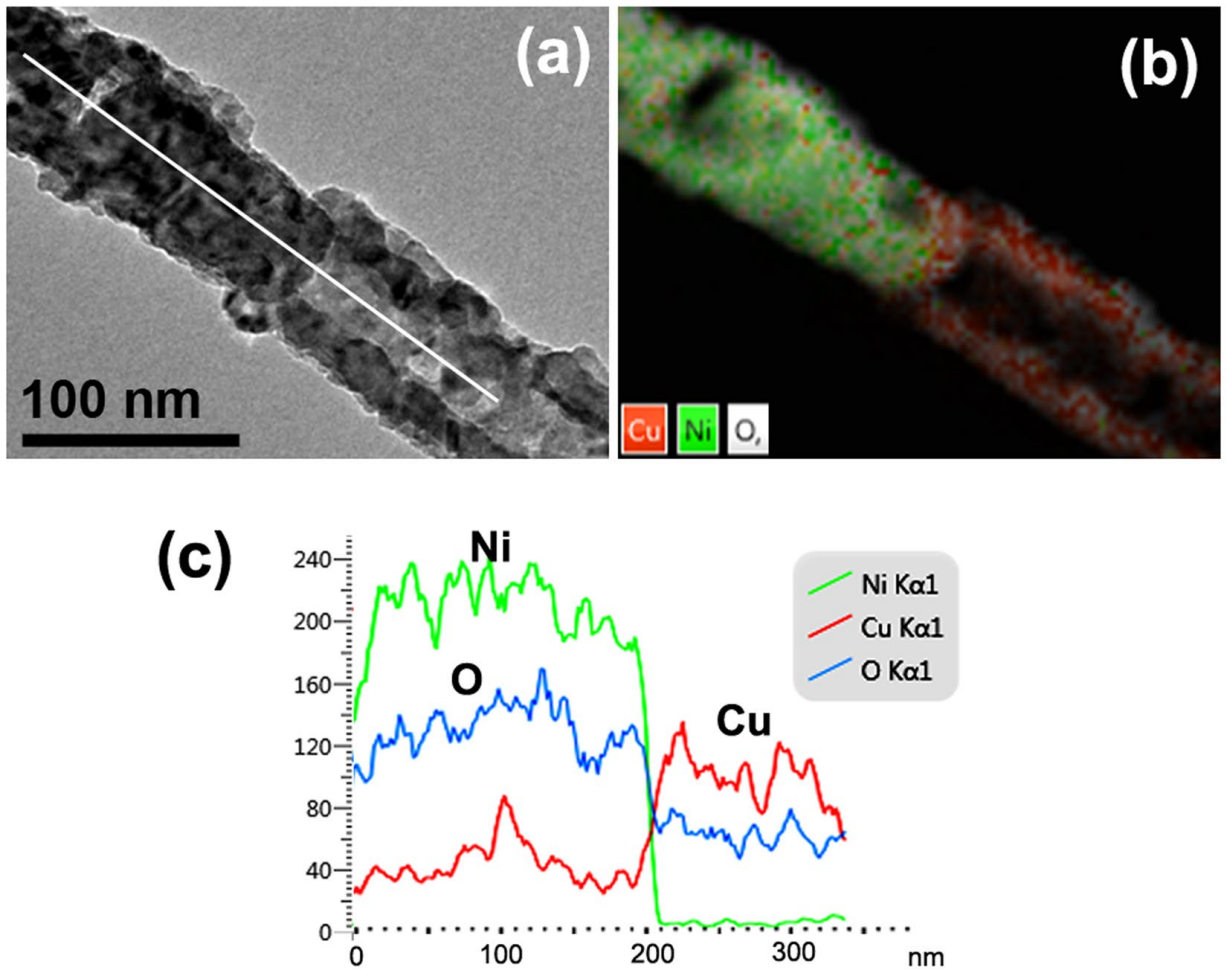
The compositional change at the interface. (**a**) TEM image of the interface between NiO and CuO; (**b**) the corresponding EDS element mapping image; (**c**) EDS line scanning image along the axis orientation as the white line shown in (**a**).

Besides the composition and morphology of each segment after annealing, we also studied the compositional change at the interface of NiO/CuO along the axis direction. Figure 11a is the TEM image of the interface between porous NiO and tubular CuO, the corresponding EDS element mapping image is shown in Fig. 11b. From the EDS element mapping image, we can see that the interface between NiO and CuO is clear, and no obvious interfacial intermix zone. Figure 11c shows EDS line scanning image along the axis orientation as the white line in Fig. 11a. Some Cu elements are observed in NiO segment, while almost no Ni element can be observed in CuO segment. A clear boundary appear in the line scanning profile, which indicates that the interface between NiO and CuO is still sharp in spite of the diffusion of Cu to Ni segment.

**Figure 12:**
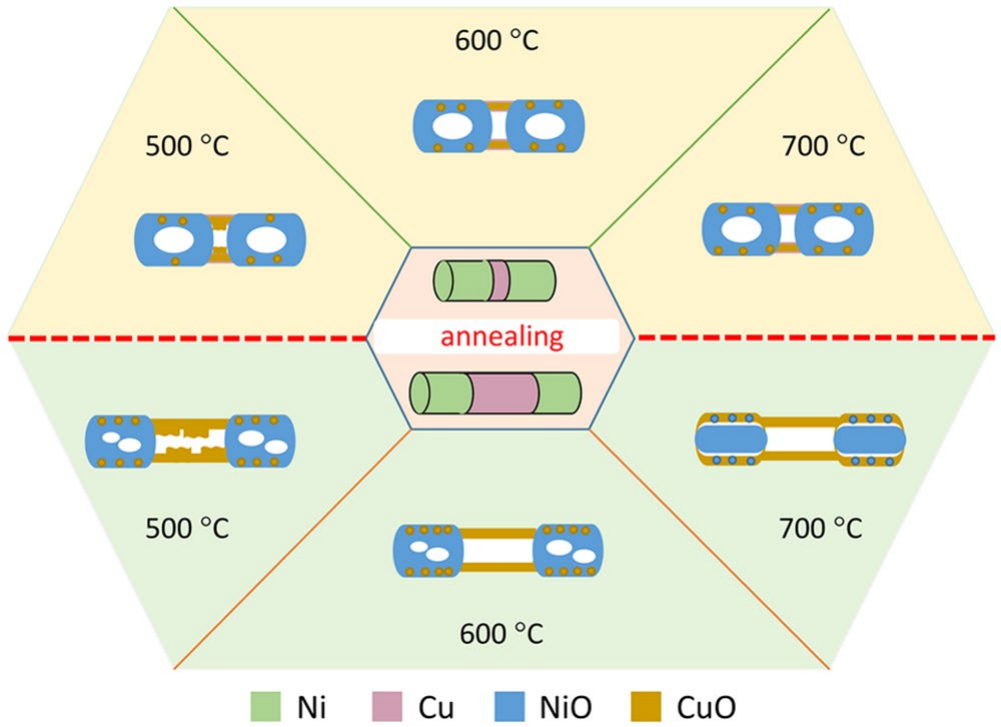
The schematic of morphology evolution of Ni/Cu nanowires after annealing at different temperatures.

The morphology evolution of Ni/Cu nanowires after annealing has been summarized in the schematic diagram as shown in Fig. 12. For Ni/Cu nanowires with relative shorter Cu segments, since the amount of Cu diffused to Ni segments is little, the morphology is changed to be the CuO tube connected with NiO hollow structure, and almost keep the same after annealing under 500 °C to 700 °C (up panel in Fig. 12). For Ni/Cu nanowires with relative longer Cu segments, the amount of Cu diffused to Ni segments is tight related with the annealing temperature, which gradually increases with temperature increasing. The morphology of Ni segments convert to porous structure after annealing at 500 °C or 600 °C, and turns into core-shell structure after annealing at 700 °C, while the morphology of Cu segments present a faint tubular structure with the nonuniform tube wall after annealing at 500 °C, and convert to a clear tube structure after annealing at 600 °C or 700 °C (bottom panel in Fig. 12). This work shows that by rationally designing the periodic length of Ni/Cu nanowires and choosing appropriate annealing temperature, the expected heterogeneous composite hollow nanostructures can be achieved. This study can be extended to the synthesis of other metal oxide heterogeneous hollow nanostructure or metal oxide wrapped precious metal particles chain structure, and may also pave the way for studying the morphology evolution of nanostructure with annealing treatment.

In summary, we have fabricated NiO-CuO one-dimensional heterogeneous composite hollow nanostructure by annealing Ni/Cu superlattice nanowires in air. After annealing at 700 °C for 2 h, Cu segments transformed to CuO nanotube and Ni segments can be controllably transferred to hollow structure or core-shell structure. By reasonably changing the relative length of Cu segments and the annealing temperature, the structural regulation was achieved. It is found that the morphology evolution of Ni segments depends on the relative diffusion amount of Cu to Ni segments. When the relative diffusion quantity is little, the oxidation behavior of Ni segments is similar to the oxidation of pure Ni, the oxidation process is mainly external oxidation, accompanying with the hollow structure formed. However, when the relative diffusion quantity is large, the oxidation of Ni segments doped with Cu atoms can be regarded as the oxidation process of Ni-Cu alloy at the nanoscale. Ni and Cu spread out and react with outside O firstly, and Cu can almost completely diffuse out and react with oxygen due to its faster diffusion rate and the very short distance. The formed Cu_2_O shell layer can offer sufficient O source to oxidize internal nano-sized Ni, which leads to Ni experiencing simultaneous external and internal oxidation, and the final oxidation product being an internal oxide of Ni lying beneath of a mixed shell layer of CuO and NiO. This work would deepen our understanding on the oxidation behaviour at nanometer scale and may guide the preparation of metal oxide heterogeneous composite hollow nanostructure.

## Methods

### Electrodeposition of Ni/Cu nanowires

Ni/Cu superlattice nanowires were prepared in the pores of AAO template by using alternated electrodeposition method with the home-made programmed device^46^. Pore size of the AAO template used here is about 50 nm. The electrolyte used for deposition of Ni consisted of 0.38 M NiSO_4_ · 6H_2_O, 0.12 M NiCl_2_ · 6H_2_O and 0.5 M H_3_BO_3_, and the pH value was adjusted to about 2.5 with 1 M H_2_SO_4_; the electrolyte used for Cu depositing contains 0.2 M CuSO_4_ · 5H_2_O and 0.5 M H_3_BO_3_ with pH value of about 2.5 adjusted by 1 M H_2_SO_4_. Ni/Cu samples with different period lengths were prepared by changing the deposition time for each element.

### Fabrication of NiO-CuO Heterogeneous Composite Hollow Nanostructure

After deposition process, the AAO template filled with Ni/Cu nanowires was corroded by NaOH solution with concentration of about 5 wt% for about 25 min to make sure that the AAO template was removed completely. Then the specimen was rinsed with distilled water and absolute ethanol for several times to clear the residues, and then the independent nanowires dispersed in absolute ethanol could be obtained by ultrasonication. At last, several droplets of the ethanol containing the Ni/Cu nanowires were dripped on the silicon wafer or silicon nitride film. After dried in air, the samples were put into the tube furnace for annealing at a various temperature from 500 °C to 700 °C in air.

### Characterization

The morphology of samples was characterized by a field emission scanning electron microscopy (FE-SEM, FEI Sirion 200); the crystal structure and chemical component were studied by transmission electron microscope (TEM, JEOL-2010), high resolution TEM (HR-TEM), and energy dispersive X-ray spectroscopy (EDS) elements line scanning and mapping scanning attached to the TEM.

## Electronic supplementary material

Supplementary information
