# Supplementary material for: Necklace-like NiO-CuO Heterogeneous Composite Hollow Nanostructure: Preparation, Formation Mechanism and Structure Control
Source: Sci Rep. 2017 Mar 10;7:144. doi: 10.1038/s41598-017-00157-0 (PMC5427851; doi:10.1038/s41598-017-00157-0)
Supplement: Supplementary file 1 — Supplementary information [file 41598_2017_157_MOESM1_ESM.doc]

Electronic Supporting Information

**Necklace-like NiO-CuO Heterogeneous Composite Hollow Nanostructure: Preparation, Formation Mechanism and Structure Control**

Shao Hui Xu, Guang Tao Fei*, Hao Miao Ouyang, Guo Liang Shang, Xu Dong Gao and Li De Zhang

Key Laboratory of Materials Physics and Anhui Key Laboratory of Nanomaterials and Nanotechnology, Institute of Solid State Physics, Hefei Institutes of Physical Science, Chinese Academy of Sciences, P. O. Box 1129, Hefei, 230031, P. R. China.

**1. Supporting Figures**

**
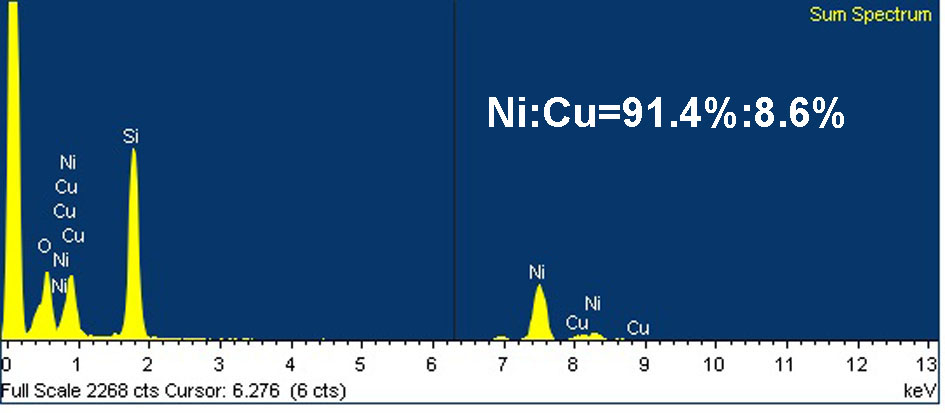
**

**Figure S1**. The sum spectrum for EDS element mapping image of the single hollow particle chain in figure 5b.


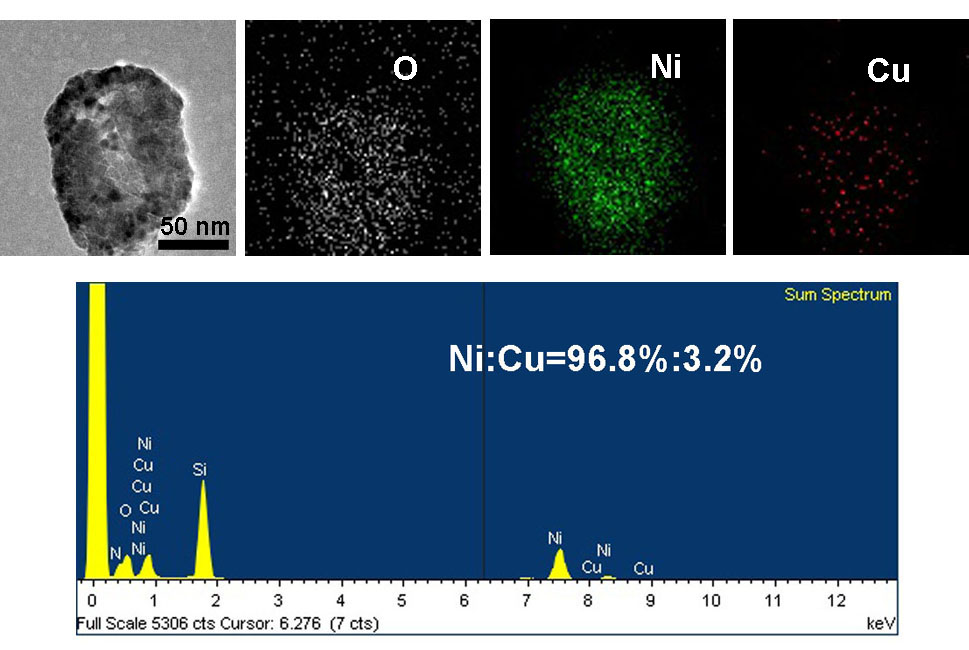


**Figure S2**. The EDS elements mapping images and the corresponding sum spectrum of the hollow particle structure.


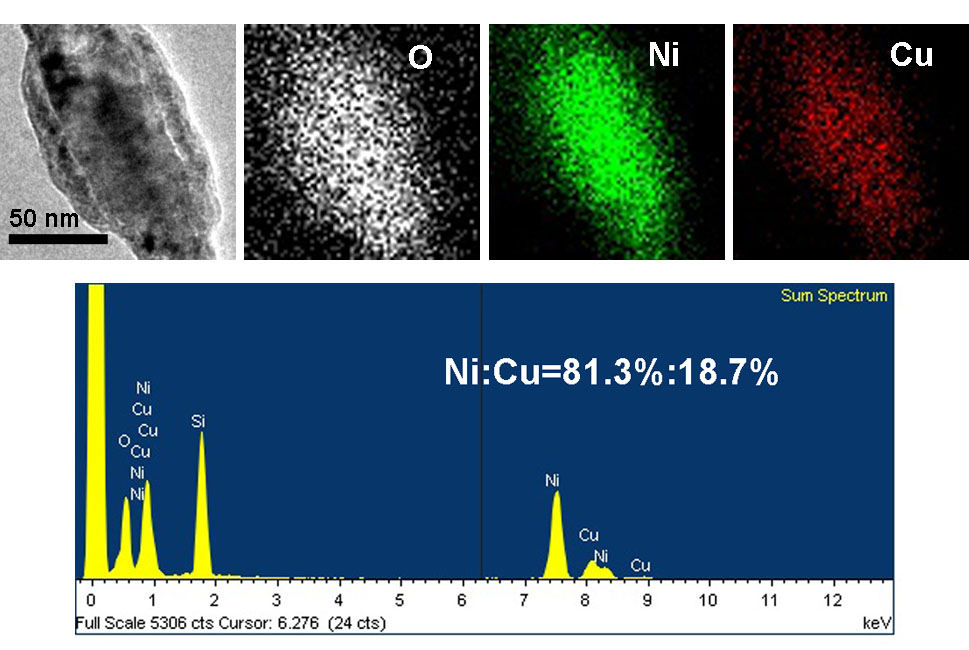


**Figure S3**. The EDS elements mapping images and the corresponding sum spectrum of the core-shell structure.

**2. Change in Gibbs free energy**

Cu_2_O+Ni=NiO+2Cu

Δ_r_G_m_^ϴ^(T)≈Δ_r_H_m_^ϴ^(298.15K)-TΔ_r_S_m_^ϴ^(298.15K)

=-72 kJ∙mol^-1^-973.15K×(-18.67×10^-3^) kJ (K∙mol^-1^)

=-72 kJ∙mol^-1^-(-18.17) kJ∙mol^-1^

**=**-53.83kJ∙mol^-1^

**Table S1 [Ref.1]**

| **T=**298.15K | Cu_2_O | Ni | NiO | Cu |
| --- | --- | --- | --- | --- |
| Δ_f_H_m_^ϴ^/kJ∙mol^-1^ | -168.6 | 0 | -240.6 | 0 |
| S_m_^ϴ^/ J (K∙mol^-1^) | 93.1 | 29.87 | 38 | 33.15 |

**References**

[1] James G. Speight, Lange's Handbook of Chemistry, Sixteenth Edition, The McGraw-Hill companies, Library of Congress Catalog Card Number 84-643191, ISSN 0748-4585, Section One, Table 1.56.
